# Green Recovery of Rosmarinic Acid via Whey Soy Protein-Mediated Foam Fractionation: Molecular Mechanisms and Enhanced Antioxidant Activity

**DOI:** 10.3390/foods15142525

**Published:** 2026-07-16

**Authors:** Yanfei Li, Run Yang, Hongjie Xiang, Zhirong Zhang, Zhijun Zhang, Nan Hu

**Affiliations:** School of Chemistry and Chemical Engineering, North University of China, Taiyuan 030051, China; yanfeili1021@163.com (Y.L.); 18839376985@163.com (R.Y.); 18696807570@163.com (H.X.); 18103426721@163.com (Z.Z.); sxzzj@163.com (Z.Z.)

**Keywords:** rosmarinic acid, foam fractionation, biosurfactants, whey soy protein

## Abstract

The sustainable isolation of nonamphiphilic phytochemicals remains a formidable challenge in biochemical engineering. In this study, a highly efficient and solvent free foam fractionation process was developed for recovering rosmarinic acid from botanical extracts. By systematically screening diverse biological surfactants, whey soy protein emerged as an exceptionally robust dual functional frother and nanoscale collector. Response surface methodology optimized the operational parameters to 850 mg/L protein concentration, pH 2.5, and a gas flow rate of 470 mL/min, yielding an outstanding target recovery of 93.08 percent alongside an enrichment ratio of 1.81. This macroscopic separation superiority was comprehensively elucidated at the molecular level through multiple spectroscopic techniques and computational modeling. Results confirmed a spontaneous static quenching complexation driven by synergistic noncovalent forces, predominantly hydrogen bonding, van der Waals interactions, π-stacking, and salt bridges. These interactions induced targeted conformational unfolding within the protein backbone, exposing hydrophobic domains that drastically elevated the thermodynamic affinity for the ascending gas–liquid interface. Furthermore, the concentrated product exhibited an antioxidant capacity enhancement exceeding 3.6 times compared to the crude extract, a result attributed to selective enrichment combined with the structural shielding effect provided by the protein macromolecule. Ultimately, this work provides critical mechanistic insights and establishes a scalable technological framework for the green purification of highly valuable botanical compounds.

## 1. Introduction

Natural compounds derived from complex plant matrices are increasingly investigated as promising alternatives or adjuncts to synthetic pharmaceuticals [1]. Among these, rosmarinic acid (RA), a phenolic ester of caffeic acid and 3,4-dihydroxyphenyllactic acid, has garnered significant attention for its potent antimicrobial, anti-inflammatory, and antioxidative properties [2,3]. Originally isolated from *Rosmarinus officinalis* L., RA is abundant in various medicinal and culinary herbs, particularly those of the Perilla species, which represent a cost-effective and scalable source [4]. The prevalence and diverse biological activities of RA underscore its therapeutic potential [5,6], generating a strong industrial impetus for the development of highly efficient, low-carbon, and sustainable separation technologies for its purification [7].

Conventionally, the recovery of RA from plant matrices relies heavily on extraction methods utilizing volatile organic solvents [8]. However, the reliance on these volatile, energy-intensive, and often toxic reagents poses significant environmental and safety challenges, impeding their large-scale industrial application in the context of modern green chemistry. This limitation raises a critical technological question: how can we isolate RA in a manner that is both economically viable and environmentally benign? Foam fractionation presents a compelling answer. As a non-solvent, bubble-mediated separation technique, it operates on the principle of the preferential adsorption of surface-active compounds onto the gas–liquid interface of rising bubbles [9,10]. Its operational simplicity, cost-effectiveness, and alignment with green chemistry principles have led to its successful application in diverse fields, from mineral beneficiation to wastewater remediation and, increasingly, the enrichment of bioactive natural products [11,12].

Despite its process advantages, the application of foam fractionation for the targeted recovery of RA is not straightforward. RA itself is a non-surface-active molecule, meaning it does not spontaneously adsorb onto the ascending bubble surfaces. This inherent property introduces a fundamental interfacial challenge: how can a non-amphiphilic target molecule be effectively captured and transported to the gas–liquid interface? The solution lies in employing a surfactant to act as a carrier or collector. While synthetic surfactants can fulfill this role, their petrochemical origins and potential ecotoxicity contradict the goal of a fully green process [12,13]. Consequently, biosurfactants have emerged as environmentally friendly alternatives. However, biosurfactants are not a monolithic group; they encompass a vast diversity of molecular structures, including glycolipids, betaines, and macromolecular proteins, each with unique physicochemical and interfacial properties [14,15].

This structural diversity raises a pivotal and more fundamental scientific question in separation engineering: How does the distinct molecular architecture of different biosurfactant classes influence their interaction mechanisms with RA and, consequently, their macroscopic separation efficacy in foam fractionation? The choice of an optimal biosurfactant is far from trivial, as its performance depends on complex molecular recognition and interfacial hydrodynamics. For instance, will the small, zwitterionic structure of a betaine facilitate efficient electrostatic binding? Will the powerful emulsifying and foaming properties of a glycolipid lead to higher enrichment? Or will the large, multi-domain structure of a plant-derived protein offer synergistic binding sites for superior complexation and interfacial stabilization? To date, a systematic comparison of these fundamentally different biosurfactant types for the recovery of a specific phytochemical like RA is lacking, leaving a critical knowledge gap in designing rational, high-efficiency separation processes.

To address this central question, this study systematically evaluates and compares three distinct classes of biosurfactants, namely a glycolipid, a betaine, and a plant-derived protein, for the separation of RA from Perilla leaf extracts via foam fractionation. By fundamentally assessing their foaming properties and separation efficiencies, we identified whey soy protein (WSP) as the most robust dual-functional frother and protein collector. While highly purified protein systems frequently employed in food processing (such as whey protein isolate or bovine serum albumin) also exhibit excellent foaming and binding properties, their high market cost limits their economic viability as sacrificial carriers in large-scale extraction. In contrast, WSP represents an abundantly available, low-cost agricultural byproduct. Its application provides comparable interfacial active sites and polyphenol-binding capabilities while significantly reducing operational costs, thereby offering a sustainable “waste-to-wealth” paradigm. Subsequently, using this top-performing macromolecule, we employ response surface methodology (RSM) to optimize key operational parameters, including biosurfactant concentration, solution pH, and gas velocity. To elucidate the underlying separation mechanisms at the molecular level, Fourier transform infrared (FTIR) and fluorescence spectroscopy are coupled with thermodynamic analyses to probe the molecular interactions and complexation between RA and the WSP collector. Furthermore, molecular docking simulations are utilized to unravel the specific non-covalent driving forces (e.g., hydrogen bonding, hydrophobic interactions) dictating this protein-polyphenol association. Finally, high-performance liquid chromatography (HPLC) and antioxidant assays are used to quantify the separation performance and biological activity preservation. This work aims to establish a comprehensive structure–performance relationship for biosurfactants in phytochemical recovery, providing fundamental insights and a practical guide for the rational design of green separation paradigms in the botanical extraction industry. Furthermore, while detailed quantitative assessments regarding exact energy consumption and carbon footprint reductions remain a critical subject for future scale-up studies, the fundamental sustainability of this process currently lies in its circular economy approach. The macromolecular collector utilized, WSP, is directly upcycled from an agricultural byproduct. Unlike petroleum-derived synthetic surfactants, WSP is fully biodegradable, ensuring that the separation process qualitatively eliminates organic solvent consumption while strictly preventing secondary environmental pollution.

## 2. Materials and Methods

### 2.1. Reagents

Low denaturation defatted soybean meal (SBM) was obtained from the Yimeng Mountain Specialty Center in Shandong, China, and was stored in a sealed container under refrigeration. Rhamnolipid (RL) was purchased from Ruijie Biotechnology Co., Ltd. (Xi’an, China). Three other biosurfactants, namely sophorolipid (SL), carboxylic betaine (BS12), and cocamidopropyl betaine (CAPB), were supplied by Youso Chemical Technology Co., Ltd. (Linyi, China). Iron (II) sulfate and Coomassie brilliant blue G250 were provided by Adamas-beta (Shanghai, China). The high-purity rosmarinic acid (RA) analytical standard with a purity exceeding 97 percent, along with various additional laboratory chemicals, was acquired from Aladdin Reagent Co., Ltd. (Shanghai, China). All experimental aqueous solutions were prepared using ultrapure water generated via a UPTC purification system (Lichenbangxi Instrument Technology Co., Ltd. (Shanghai, China)).

### 2.2. Extraction of RA

To extract the target compound, dried Perilla leaves were mechanically ground to a pulverized state. An initial recovery procedure was executed by modifying a previously reported technique by Hu et al. [16]. These specific parameters (90 °C for 80 min) were adapted from this previously validated methodology, which ensured efficient leaching of the target compound while avoiding the severe thermal degradation commonly associated with more extreme processing. Specifically, 5.0 g of the milled plant material was suspended in 200 mL of purified water. This heterogeneous mixture was subjected to thermal extraction in an HH-ZK1 heated water bath (Yuhua Instrument Co., Ltd. Gongyi, China) maintained at 90 °C for a duration of 80 min. Post-heating, the mixture was filtered through triple-layered medical gauze to remove macroscopic solid residues, followed by centrifugation of the filtrate at 4000 rpm for 10 min. The clarified upper liquid fraction subsequently functioned as the primary feedstream for the downstream foam separation column.

### 2.3. Whey Soy Protein (WSP) Preparation

The WSP collector was synthesized utilizing the aforementioned SBM material. Relying on procedures outlined in our previous study [17], the dry SBM was first milled thoroughly. Subsequently, 1.0 g of this powder was dispersed in 17 mL of deionized water, followed by adjusting the pH of the suspension to 9.0 using alkaline solutions. The mixture was continuously stirred using a thermostatic magnetic stirrer (Guoyu Instrument Manufacturing Co., Ltd., Changzhou, China) at 51 °C for 48 min. Following a 10 min primary centrifugation at 4000 rpm, the supernatant was collected and its pH was adjusted to 4.5. A subsequent 15 min centrifugation at the identical rotational speed was performed to effectively precipitate and eliminate any insoluble matter under these acidic conditions. Finally, the pH of the secondary supernatant was neutralized, the moisture was removed via lyophilization, and the resulting WSP powder was preserved at 4 °C for subsequent usage. Furthermore, proteomic composition analyses confirmed that the relative contents of its primary functional components, Kunitz trypsin inhibitor (KTI) and Bowman–Birk inhibitor (BBI), account for approximately 40% and 30% of the total protein content, respectively [18].

### 2.4. Foaming Property

Prior to the fractionation experiments, the fundamental foaming properties of the five selected biosurfactants and the RA extract were systematically evaluated. Specifically, a modified Ross–Miles method was employed to measure both the initial foam height and the foam half-life. A detailed characterization of the foam structure is provided in Supplementary Section S2. Furthermore, the surface tension of the solutions was quantified using an automatic interfacial tensiometer (JK99B, Zhongchen Digital Technology Equipment Co., Ltd., Shanghai China).

### 2.5. Foam Fractionation of RA

For comprehensive diagrams depicting the custom-built fractionation rig and the detailed standard operating procedures for the bubbling process, please consult Section S1. During the main foam fractionation experiments, the five biosurfactants functioned dually as protein collectors and frothers. The separation performance of the system was systematically quantified using two primary metrics, namely the recovery percentage (*R_RA_*) and the enrichment ratio (*E_RA_*) of *RA*, calculated via Equations (1) and (2), respectively. To gain deeper insights regarding the interfacial thermodynamic and fluid dynamics, we calculated two additional hydrodynamic parameters: fractional liquid holdup (*ε*_out_) and molecular surface excess (*Γ*). Section S3 contains the exact mathematical derivations supporting these particular calculations.

(1) $$R_{RA}=\frac{C_{0}Q_{0}-C_{r}Q_{r}}{C_{0}Q_{0}}\times 100\%$$

(2) $$E_{RA}=\frac{C_{0}Q_{0}-C_{r}Q_{r}}{C_{0}(Q_{0}-Q_{r})}$$

The mathematical framework for *RA* mass transfer analysis was established using the following nomenclature:

*C*_0_ (mg/L): Initial *RA* concentration in the extraction solution prior to fractionation;

*C_r_* (mg/L): Residual *RA* concentration in the retentate phase post-foam fractionation;

*Q*_0_ (mL/min): Volumetric flow rate of the feedstock entering the fractionation system;

*Q_r_* (mL/min): Volumetric flow rate of the retentate stream exiting the separation unit.

### 2.6. Characterization and Detection

To investigate the microscopic structural features alongside atomic composition variations between pure WSP and the resulting WSP-RA agglomerates, we utilized an Apreo 2S scanning electron microscope (SEM, Thermo Scientific, Waltham, MA, USA) equipped with an integrated energy dispersive X-ray spectroscopy detector. To identify the functional groups on the material surfaces, Fourier transform infrared (FTIR) spectra were recorded over a wavenumber range of 400–4000 cm^−1^ using a spectrometer (Vector-22, Baden-Württemberg, Bruker, Germany). Additionally, the zeta potentials of the samples were measured with a zeta potentiometer (JS94K2, Powereach, Shanghai, China). To eliminate background UV interference from other plant pigments in the crude extract, a specific metal-complexation colorimetric assay was employed. RA reacts with Iron (II) sulfate to form a stable colored complex, and its concentration was subsequently determined at its maximum absorption wavelength of 568 nm using a UV–visible spectrophotometer (UV-5200PC, Metash Instruments Co., Ltd., Shanghai, China) according to the method described by Li et al. [19]. Detailed procedures for the fluorescence measurements and the evaluation of antioxidant activities including DPPH and ABTS assays are provided in Supplementary Sections S4 and S5, respectively.

### 2.7. Molecular Docking

To conduct computational molecular docking simulations, AutoDockTools version 1.5.6 was employed. We acquired the crystal configuration of Kunitz trypsin inhibitor (KTI), a major structural component of WSP, assigned with the PDB code 3NPO through the RCSB Protein Data Bank. Subsequent preparation involved removing aqueous molecules and extraneous ligands utilizing the PyMol software suite version 2.2.0. For the target ligand, the three-dimensional coordinates representing RA were retrieved directly from PubChem. These structural files were subsequently converted into the requisite PDBQT format. Standard default settings were applied to all remaining simulation parameters. The algorithm generated ten top-ranked conformational poses. From this generated pool, the most optimal spatial arrangement was determined by evaluating its structural alignment alongside the corresponding binding energy. Final visualization of these interactive complexes was achieved via PyMol.

### 2.8. Statistical Analysis

Every experiment was conducted in three independent replicates, with the standard errors visually denoted by error bars. Statistical variance and data significance were evaluated utilizing Student’s *t*-test via Microsoft Excel, establishing a statistical significance threshold of *p* ≤ 0.05. All graphical representations were plotted utilizing Origin 2023 software. Furthermore, the formulation of the Box–Behnken design (BBD) matrix and its associated statistical evaluations were executed using Design-Expert software (version 13; Stat-Ease, Inc., Minneapolis, MN, USA).

## 3. Results

### 3.1. Foam Property of Biosurfactants

#### 3.1.1. Effect of Biosurfactants Concentration

**Figure 1:**
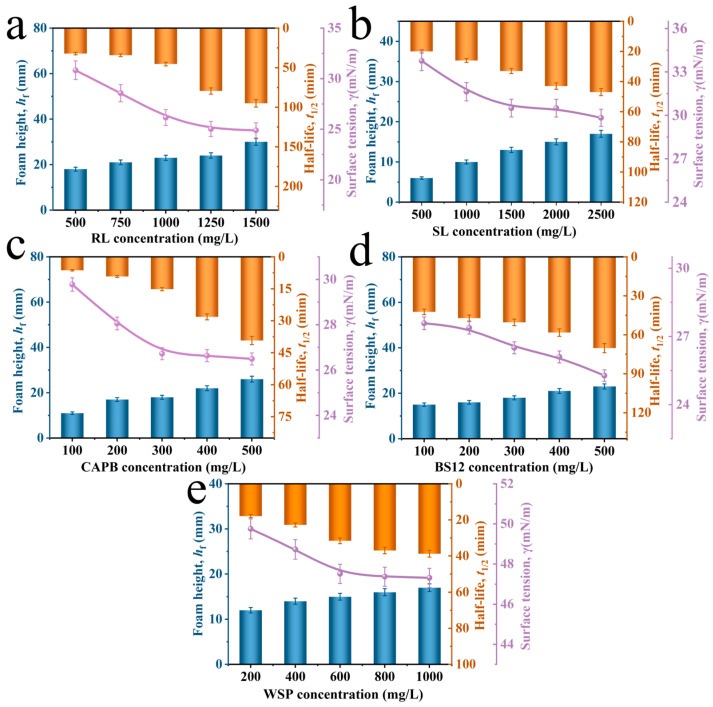
(**a**–**e**) Surface tension, foam height and half-life of WSP with 200 mg/L RA extracting solution stabilized foams (pH 7.0) as a function of the concentrations of the five biosurfactants (RL, SL, CAPB, BS12 and WSP).

**Figure 2:**
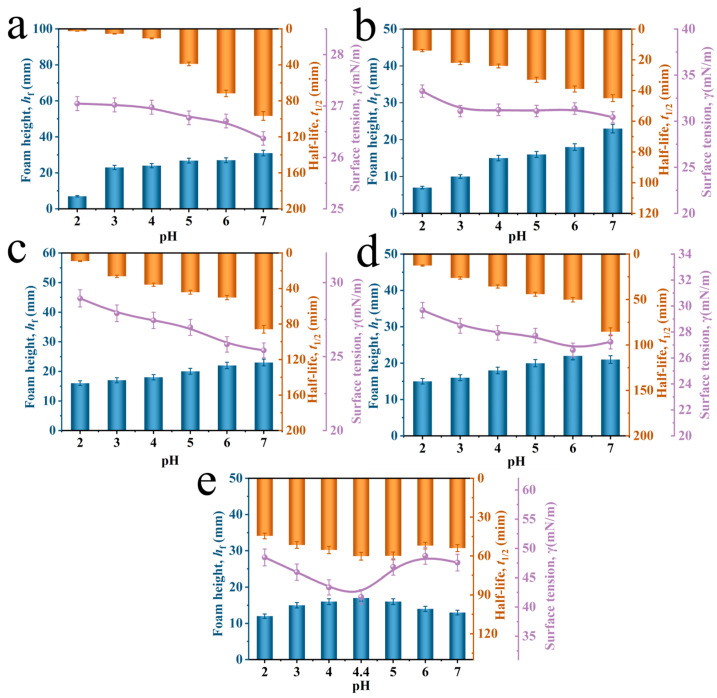
(**a**–**e**) Surface tension, foam height and half-life of the five biosurfactants with 200 mg/L RA extracting solution stabilized foams as a function of pHs of the five biosurfactants (*C*_RL_ = 1250 mg/L, *C*_SL_ = 2500 mg/L, *C*_CAPB_ = 400 mg/L, *C*_BS12_= 300 mg/L and *C*_WSP_ = 800 mg/L).

The foamability of the RA extract solution at a concentration of 200 mg/L combined with five biosurfactants (rhamnolipid (RL), sophorolipid (SL), cocamidopropyl betaine (CAPB), carboxylic betaine (BS12), and whey soy protein (WSP)) was assessed by measuring surface tension, foam height, and half life as a function of biosurfactant concentration and pH. The fundamental foaming properties of these systems under varying concentrations and pH conditions (2.0 to 7.0) were systematically recorded and are depicted in Figure 1 and Figure 2.

Given the distinct physicochemical nature of the evaluated biosurfaurectants, variable concentration regimes were applied. Within lower concentration intervals ranging from 100 to 400 mg/L, the foaming capability and half life of RL and SL proved inadequate; thus, their initial testing concentration was elevated to 500 mg/L. As visually presented in Figure 1a–e, the foam height and half life exhibited a similar dose dependent trend across all five biosurfactants. Specifically, these foaming metrics initially increased and subsequently reached a plateau as the biosurfactant concentration escalated. Furthermore, Figure 1a–e demonstrate that CAPB achieved the highest foam height and the longest half life at a concentration of 500 mg/L.

#### 3.1.2. Effect of Solution pH

The environmental pH exerts a profound impact on the foaming performance of the biological surfactants, as comprehensively illustrated in Figure 2a–e. Because RA is prone to degradation under alkaline conditions, the investigated pH spectrum was constrained between 2.0 and 7.0. For the small-molecule surfactants including RL, SL, CAPB, and BS12, both the height and half life of the foam increased proportionately with an elevation in pH. Conversely, the macroscopic foaming behavior of WSP displayed a distinctly nonlinear trend. Starting from highly acidic conditions, the foam height and half life of WSP initially increased, peaked at a pH of approximately 4.4, and subsequently declined as the alkalinity further increased. These macroscopic patterns are fundamentally corroborated by the pH dependent surface tension measurements, wherein the optimal foaming metrics perfectly coincided with the lowest surface tension values.

#### 3.1.3. Micromorphology of Foams

**Figure 3:**
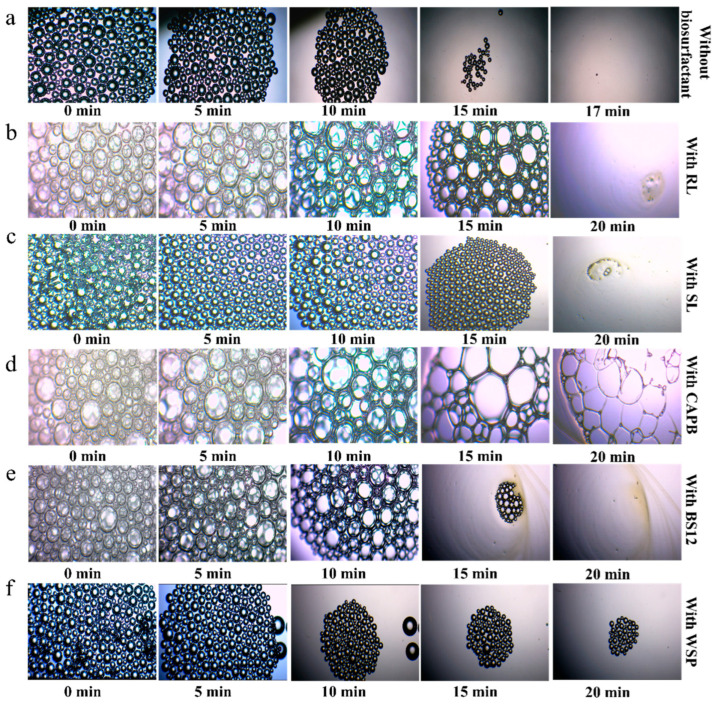
(**a**–**f**) Microscopic images detailing the temporal evolution of the foam morphology stabilized by the RA extracting solution, both with and without the addition of the five biosurfactants (*C*_RL_ = 1250 mg/L, *C*_SL_ = 2500 mg/L, *C*_CAPB_ = 400 mg/L, *C*_BS12_= 300 mg/L and *C*_WSP_ = 800 mg/L).

Observing temporal changes in bubble morphology provides a reliable visual metric for evaluating the foaming characteristics of rosmarinic acid (RA) extraction systems. Figure 3a–f illustrates microscopic structural differences between systems lacking surface-active agents versus those containing them. Parameters including individual bubble geometry, diameter distribution, and overall packing density directly dictate macroscopic stability [20]. In the absence of additives, generated bubbles collapsed entirely before twenty minutes elapsed. Conversely, incorporating biological surfactants preserved structural integrity well beyond this timeframe. Micrographs featuring RL, SL, CAPB, and BS12 (Figure 3b–e) reveal a heterogeneous size distribution, prominent structural overlap, and exceptionally thin lamellae. In striking contrast, the presence of WSP significantly delayed bubble rupture events and effectively impeded structural coarsening.

### 3.2. Effectiveness of RA Separation Using Different Biosurfactants

#### 3.2.1. Effect of Biosurfactants Concentration

**Figure 4:**
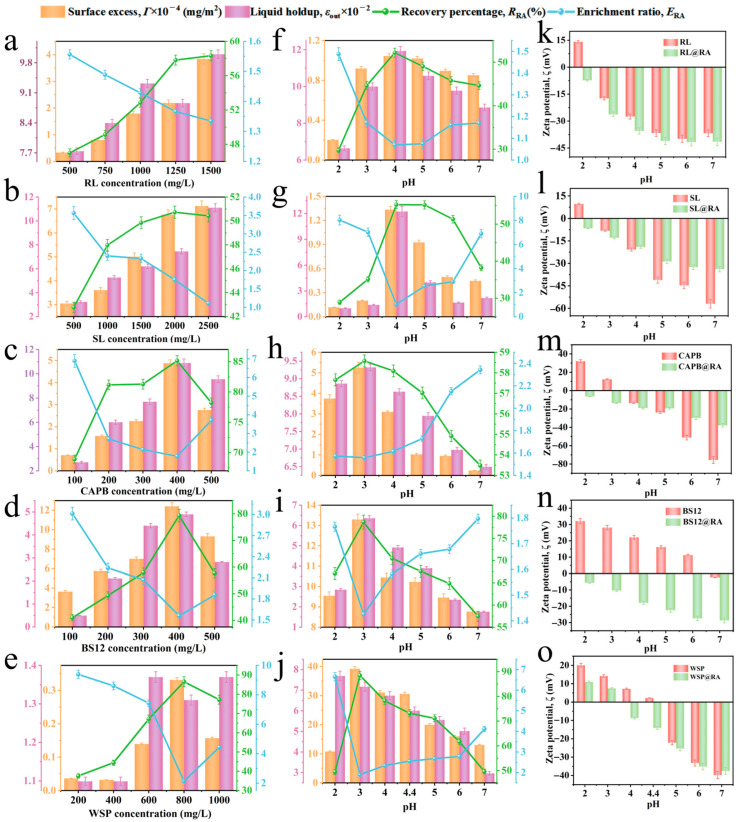
Effects of five biosurfactants concentration on the recovery percentage (%), enrichment ratio, surface excess (×10^−4^ mg/m^3^) and liquid holdup (×10^−2^) for the treatment of 200 mg/L RA extracting solution (**a**–**e**), respectively; effects of pH on the recovery percentage (%), enrichment ratio, surface excess (×10^−4^ mg/m^3^) and liquid holdup (×10^−2^) for the treatment of 200 mg/L RA extracting solution (**f**–**j**), respectively; effect of pH on the zeta potential of five biosurfactants with RA extracting solution (**k**–**o**).

We comprehensively evaluated how varying additive concentrations impacted overall fractionation efficiency, presenting these quantitative findings across Figure 4a–e. Both the enrichment ratio (*E_RA_*) and overall recovery percentage (*R_RA_*) corresponding to the target molecule displayed significant dose-dependent fluctuations. Systems utilizing CAPB, BS12, and especially WSP showcased remarkable sensitivity toward concentration alterations. As visually confirmed in Figure 4e, a substantial enhancement in the recovery ratio from approximately 37.6% to a peak of 86.5% was observed as the WSP concentration rose from 200 mg/L to 800 mg/L. Based on the maximum recovery achievements, the separation efficiencies of the diverse biosurfactants followed the descending order: WSP ≈ CAPB > BS12 > RL > SL. Consequently, the optimal collector concentrations were precisely determined as 1250 mg/L for RL, 2000 mg/L for SL, 400 mg/L for CAPB, 400 mg/L for BS12, and 800 mg/L for WSP. Furthermore, as universally observed across Figure 4a–e, an inevitable trade-off materialized at elevated biosurfactant concentrations: a monotonic decline in RA enrichment ratios coupled with a concomitant rise in liquid holdup rates (*ε*_out_).

#### 3.2.2. Effect of pH

The dynamic dependence of RA foam fractionation on pH was systematically evaluated across a range of 2.0 to 7.0, with the results comprehensively illustrated in Figure 4f–j. A characteristic bell-shaped profile is consistently observed for RA recovery, surface excess, and liquid holdup as pH increases, whereas the enrichment ratio inevitably exhibits the inverse parabolic trend due to the aforementioned liquid–drainage trade-offs. A critical appraisal of Figure 4f–j confirms that the glycolipid-based biosurfactants yielded the relatively poorest separation efficiencies (RL peaking at *R* ≈ 52% at pH 4; SL at *R* ≈ 55.2% at pH 4). The betaine-type biosurfactants displayed intermediate to high capabilities (CAPB peaking at *R* ≈ 58% at pH 3; BS12 demonstrating robust affinity with *R* ≈ 78% at pH 3). Distinctly, when WSP was employed as the collector, the separation efficiency reached an exceptional maximum, approaching 88–90% at pH 3.

**Figure 5:**
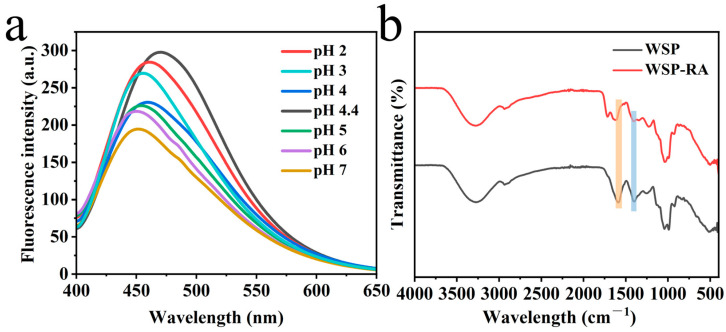
pH on extrinsic fluorescence spectra of WSP with RA (**a**), FTIR spectra of WSP and WSP-RA (**b**).

To further elucidate the underlying mechanism responsible for the superior performance of WSP, extrinsic fluorescence spectroscopy was utilized to evaluate WSP surface properties across varying acidity levels [21,22]. As illustrated in Figure 5a, the emission spectra exhibited a maximum intensity at pH 4.4. Beyond this threshold, increasing the alkalinity caused a substantial drop in peak height. Process performance metrics fluctuated alongside these structural shifts; making the solution more alkaline initially lowered RA enrichment ratios before triggering a slight recovery. In direct contrast, volumetric liquid holdup experienced an initial surge prior to experiencing a steady decline.

### 3.3. Box–Behnken Design Optimization of Operational Parameters

Based upon preliminary single-factor trials, central point conditions (designated as zero levels) for the response surface methodology were defined. Specifically, these baselines incorporated an initial WSP concentration of 800 mg/L (variable *A*), a solution acidity of pH 3.0 (variable *B*), along with a fixed volumetric gas flow rate measuring 400 mL/min (variable *C*). As documented within Supplementary Table S1, recorded experimental outcomes demonstrated R fluctuating between 73.76% and 92.99%. Concurrently, the calculated E spanned from 1.84 up to 2.62.

Subsequent mathematical modeling yielded second-order polynomial formulas that successfully characterize how independent operating variables influence target responses. Equations (3) and (4) visually represent these interactive mathematical relationships. Comprehensive analysis of variance (ANOVA) metrics concerning both developed mathematical frameworks are provided across Supplementary Tables S2 and S3. Both established equations demonstrated robust statistical validity, a fact supported by determination coefficients (*R*^2^) reaching 0.9569 regarding recovery and 0.9508 concerning enrichment. Furthermore, corresponding lack-of-fit evaluations yielded non-significant probabilities (*p* = 0.1077 and *p* = 0.0561, respectively). Furthermore, the adequate precision values, which measure the signal-to-noise ratio, were determined to be 12.0738 for the recovery (*R*) model and 10.8982 for the enrichment ratio (*E*) model. Both values are well above the desirable threshold of 4.0, confirming that the developed mathematical frameworks possess adequate precision and high fidelity for navigating the design space and predicting actual physical performance. This statistical observation verifies that our theoretical models reliably predict actual physical performance during the separation process [23].

Evaluating individual factor impacts revealed that gas flow rate exerted the most dominant effect upon both target metrics. System acidity functioned as the secondary influential parameter. Statistical evaluation further emphasized that quadratic components played a crucial role in the process. For *R*, synergistic interactions occurred primarily between variables *A* and *B*, alongside *B* and *C*. Regarding the *E*, notable combined effects manifested exclusively between pH and airflow (*B* and *C*), accompanied by strong quadratic influences. Conversely, any remaining cross-variable interactions yielded *p*-values exceeding 0.05 [24]. Consequently, they exerted negligible mechanistic influence over separation efficacy under the currently evaluated operational limits.

(3) $$\begin{array}{l} R=-173.16349+0.155458A+69.99382B+0.456589C\\ -0.015878AB-0.000055AC-0.050149BC\\ -0.000051A^{2}-6.53171B^{2}-0.000283C^{2} \end{array}$$

(4) $$\begin{array}{l} E=9.74853-0.004311A-2.81640B-0.008364C\\ -0.000028AB+1.57664*10^{-6}AC+0.001747BC\\ +2.11258*10^{-6}A^{2}+0.375691B^{2}+7.52817*10^{-7}C^{2} \end{array}$$

**Figure 6:**
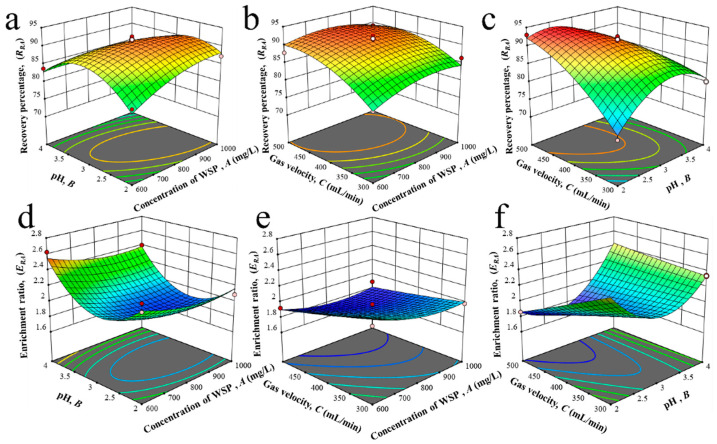
(**a**–**f**) Response surfaces illustrating the combined effects of three independent variables (WSP concentration, pH value, and gas velocity) on two response variables (*R* and *E*).

3D response surface graphs depicted within Figure 6 elucidate how varying operating parameters interactively influence the separation efficiency (specifically, the recovery and enrichment) of the target molecule. Upon examining panels (a) and (c), it becomes evident that combined variations involving initial WSP dosage alongside either system acidity or volumetric airflow critically dictate the recovery percentage. Regarding the enrichment factor, panel (f) reveals pronounced synergistic effects occurring exclusively between solution pH and gas flow rate.

Through the numerical optimization capabilities provided by the Design-Expert suite, the theoretical ideal criteria required to maximize RA isolation were calculated. The program recommended an initial surfactant concentration of 843.69 mg/L, combined with an acidic environment (pH 2.42) and a sparging rate equal to 474.56 mL/min. Applying this exact parameter combination yielded mathematically forecasted values of 93.36% for recovery alongside an enrichment ratio approximating 1.78. Recognizing typical equipment precision limits encountered during actual laboratory practice, these theoretically derived figures required slight modifications. Consequently, validation runs utilized accessible target setpoints: 850 mg/L WSP, an adjusted pH measuring 2.5, and a standardized bubbling flow of 470 mL/min. Empirical verification consisted of triplicate batch trials executed employing these modified settings. The resulting experimental averages demonstrated a recovery reaching 93.08% coupled with a 1.81 concentration factor. The minimal deviations observed between empirical data and software-generated forecasts effectively validate the established quadratic framework.

### 3.4. Mechanisms Underlying the Complexation Between RA and WSP Collector

#### 3.4.1. Morphological and Structural Reorganizations

**Figure 7:**
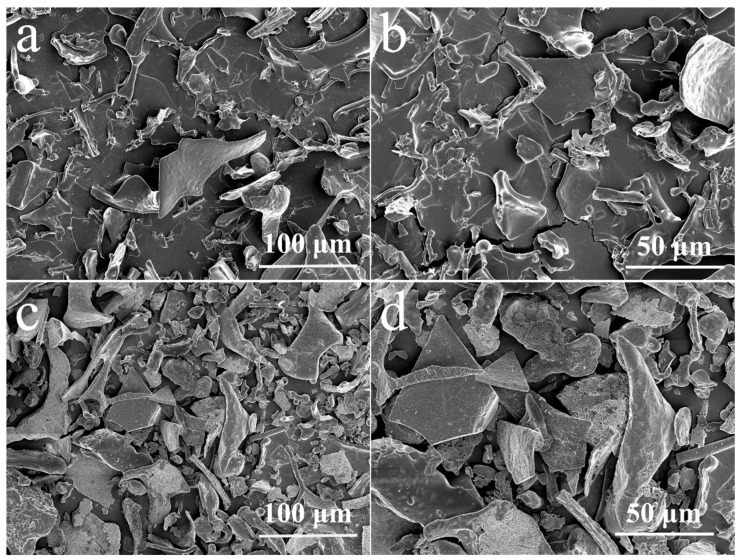
SEM image of WSP (**a**,**b**) and WSP-RA (**c**,**d**).

Morphological and elemental analyses revealed significant structural reorganization in WSP following RA interaction. SEM characterization demonstrated that native WSP exhibited characteristic flaky, irregular morphology with strip-like features (Figure 7a,b), aligning with previous reports on similar proteinaceous materials [25]. Remarkably, RA complexation induced dramatic topological changes, transforming the architecture into compact, uniform structures with smooth, integrated surfaces (Figure 7c,d). To further quantify these morphological transitions, a statistical particle size distribution analysis was performed (Figure S3). The pure WSP exhibited an average particle diameter of 18.82 ± 10.22 μm. Upon binding with RA, the average diameter of the WSP-RA complexes increased to 22.80 ± 10.73 μm. This quantitative size expansion and the shift in the distribution profile physically support the qualitative SEM observations, confirming that the non-covalent cross-linking between the targeted polyphenols and the protein matrix induces structural aggregation and the formation of larger, more integrated complex micro-particles.

To further comprehend this interaction phenomenon, energy-dispersive X-ray spectroscopy (EDS) was conducted alongside elemental mapping (Figure S2). While the spatial arrangement of elements remained uniform across both samples, their relative abundances shifted noticeably. The unmodified protein presented a standard compositional profile consisting largely of carbon (56.76%) and oxygen (40.97%), alongside minor fractions of nitrogen (1.63%), phosphorus (0.51%), and sulfur (0.13%) (Figure S2a). Following the addition of RA (Figure S2b), the carbon proportion decreased substantially to 52.20%. Simultaneously, the respective quantities of oxygen, nitrogen, phosphorus, and sulfur rose to 44.49%, 2.19%, 0.87%, and 0.25%.

To elucidate the specific chemical moieties driving these morphological and elemental transformations, Fourier transform infrared (FTIR) spectroscopy was subsequently employed. This analytical technique effectively tracks microenvironmental shifts within the macromolecular network. Spectra comparing the raw protein and the newly formed composite are presented in Figure 5b. A distinct, wide peak spanning 3000 to 3700 cm^−1^ attributed to the stretching vibrations of hydroxyl moieties [26]. For analyzing secondary conformations, the amide I (C=O stretching, 1600–1700 cm^−1^) and amide II (coupled C-N stretching and N-H bending, 1450–1600 cm^−1^) regions provide the most critical insights [27]. When evaluating the comparative data, a remarkable displacement occurred within the amide I region. Specifically, the absorption peak migrated from 1603.04 cm^−1^ in the native state to a higher frequency of 1624.73 cm^−1^ in the bound complex. Meanwhile, the amide II region exhibited no observable deviations.

#### 3.4.2. Fluorescence Quenching and Thermodynamic Driving Forces

**Figure 8:**
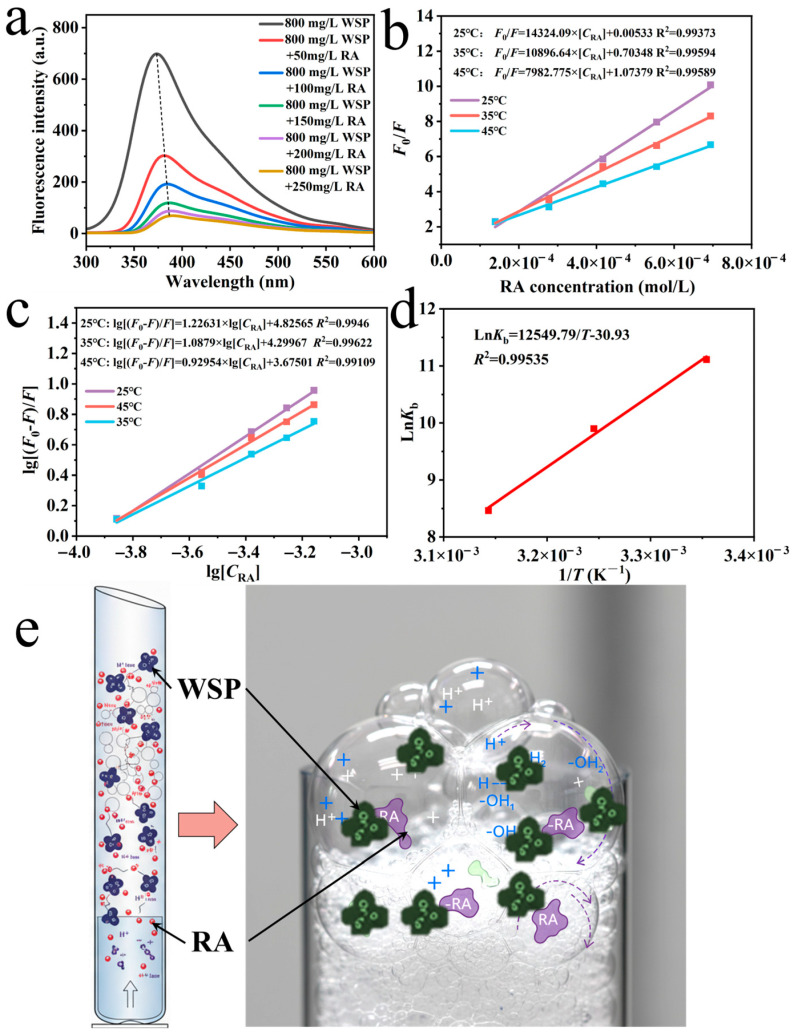
(**a**) The fluorescence spectra of the WSP–RA system at 298.15 K, *λ*_ex_ = 280 nm. The concentration of WSP was 800 mg/L, and the concentration of RA (0–250) were 0, 50, 100, 150, 200, 250 mg/L, respectively. (**b**) Stern–Volmer plot of WSP quenching by RA; (**c**) plot of lg[(*F*_0_ − *F*)/*F*] as a function of lg[*C_RA_*] at 298.15 K; (**d**) Van’t Hoff plot of the interaction between WSP and RA; (**e**) schematic description of the mechanism underlying the foam fractionation of RA using WSP as the frother and protein collector.

Evaluating the structural microenvironment surrounding specific chromophores is frequently achieved through fluorometric analytical techniques [28]. Within the present investigation, we utilized signal attenuation assays to probe the binding affinities coupling the surfactant and the target macromolecule. This was accomplished by tracking variations in the natural emission of internal tryptophan fluorophores. Illustrated within Figure 8a, the untreated biomolecule displayed an intense photoluminescent signal peaking around 370 nm when excited at a wavelength of 280 nm. This initial peak originates predominantly from buried tryptophan residues. Introducing the collector caused a gradual decline in signal magnitude, which coincided with a distinct bathochromic (red) shift of the maximum emission wavelength.

(5) $$\frac{F_{0}}{F}=K_{SV}\times [C_{RA}]+1$$

(6) $$\ln K_{B}=-\frac{\Delta H}{RT}+\frac{\Delta S}{R}$$

(7) $$\mathrm{lg}\frac{F_{0}-F}{F}=\mathrm{lg}K_{B}+n\mathrm{lg}[C_{RA}]$$

(8) $$\ln K_{B}=-\frac{\Delta H}{RT}+\frac{\Delta S}{R}$$

Evaluating the exact mode of this signal reduction required constructing Stern–Volmer profiles across various thermal conditions (Figure 8b). Utilizing Equation (5) yielded linear regressions with exceptional accuracy (*R*^2^ > 0.99). Calculating the bimolecular rate parameter (*K*_q_) provides critical evidence for distinguishing between these two mechanisms. As documented in Table 1, the calculated kinetic parameters substantially surpassed the established theoretical threshold for diffusion-controlled collisional processes (2 × 10^10^/[M·S]). Moreover, computing the stoichiometric coefficient (*n*) returned a value extremely close to unity.

To ascertain the underlying energetic drivers dictating this attachment, thermodynamic variables were deduced via Equations (7) and (8). These computed metrics are summarized in Table 1. Across all tested scenarios, the calculated Gibbs free energy (Δ*S*) remained consistently below zero.

#### 3.4.3. Computational Validation via Molecular Docking

To elucidate precise spatial alignments and molecular-level driving forces governing the surfactant-protein association, in silico modeling was conducted. Based on our recent proteomic profiling [18], the Kunitz trypsin inhibitor (KTI) and the Bowman–Birk inhibitor (BBI) constitute the primary structural fractions within the WSP isolate. Consequently, these two distinct macromolecules served as representative models for computational screening against the target ligand [29]. Visualizations and quantitative metrics for these simulated networks, utilizing specific structural models (1BBI and 1AVU), are documented within Figure 8 and Table 2. Thermodynamic computations revealed highly favorable docking scores of −3.52 kcal/mol for the KTI-collector assembly and −4.18 kcal/mol for the corresponding BBI aggregate [30,31].

**Figure 9:**
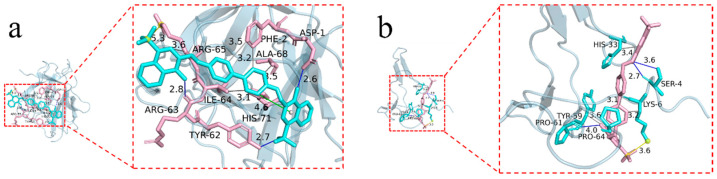
Molecular docking of KTI (**a**) and BBI (**b**) with WSP.

Detailed spatial analyses highlighted distinct non-covalent interaction networks. For the KTI model, the collector established robust hydrogen-bonded linkages with specific key amino acids: ASP-1, TYR-62, and ARG-63. The respective atomic distances for these linkages measured 2.10, 1.78, and 1.87 Å. Additionally, non-polar structural clustering occurred adjacent to residues PHE-2, ILE-64, ARG-65, and ALA-68 (Figure 9a). This localized pocket was further stabilized via π-stacking involving HIS-71, complemented by a stabilizing ionic salt bridge connecting to ARG-65. Conversely, evaluating the BBI interface identified hydrogen bonding pathways linking the ligand to SER-4 (1.94 Å) and PRO-61 (3.10 Å). Substantial hydrophobic contacts emerged near residues LYS-6, HIS-33, TYR-59, and PRO-64 (Figure 9b). Furthermore, unique ionic bridging was documented exclusively at the LYS-6 position.

### 3.5. Antioxidant Capacity

Assessing this specific biochemical trait is routinely accomplished via the DPPH methodological framework. Despite lacking a conventional oxidizable target, this rapid spectrophotometric approach remains a highly reliable standard for quantifying radical quenching dynamics. Comparative profiles detailing the neutralization capacities of the raw aqueous mixture versus the recovered foam condensate are illustrated within Figure S4. Following the bubbling separation procedure, the concentrated fraction demonstrated a remarkable amplification in radical-quenching efficacy compared to the untreated feed solution. This performance upgrade proved particularly pronounced at relatively dilute dosages. Specifically, at testing increments of 50, 100, and 150 mg/L, the purified collector exhibited radical-scavenging capacities measuring 3.6-fold, 3.2-fold, and 2.4-fold higher than the baseline crude extract, respectively.

## 4. Discussion

### 4.1. Foam Property of Biosurfactants

Foamability and foam stability are critical determinants of foam fractionation performance. From a thermodynamic perspective, the introduction of biological surfactants facilitates their spontaneous migration to the gas and liquid interface, leading to a significant decrease in the surface tension of the RA extract solution. Once the concentration approaches the critical micelle concentration, the interface becomes saturated with surfactant molecules, and any further reduction in surface tension becomes marginal, which fundamentally explains the observed plateau in foaming properties. This superior performance is directly attributed to its exceptional capability to rapidly lower interfacial free energy.

The unique, bell shaped dependency of the WSP system is a classic characteristic of amphoteric biopolymers. Specifically, the observed performance peak at pH 4.4 aligns with the isoelectric point of WSP. At this specific hydrogen ion concentration, the net electrical charge of the proteinaceous molecules approaches zero, which effectively neutralizes intermolecular electrostatic repulsion. This uncharged state promotes the tightest molecular packing at the gas and liquid interface. Consequently, WSP dynamically folds and adsorbs to construct a highly cohesive, viscoelastic interfacial film, which dramatically maximizes both foamability and structural stability compared to other pH regimes where the biopolymers carry strong repulsive charges.

This fragile microstructural configuration established a highly connected network of Plateau borders, which accelerated the kinetics of liquid drainage and ultimately led to structural destabilization after twenty minutes. This robust longevity stems from superior interfacial rheology. Upon adsorption, WSP macromolecules unfold to construct rigid, viscoelastic protein layers capable of retarding lamellar thinning and resisting mechanical perturbations [32]. Consequently, the driving force for gas diffusion across the liquid film is minimized, and Ostwald ripening is effectively suppressed, a mechanism that aligns perfectly with previous findings by Hu et al. [33]. Overall, WSP emerged as the most exceptional frother candidate, producing a homogeneous, tightly packed, and resilient microbubble matrix perfectly suited for the subsequent mass transfer and separation of RA.

### 4.2. Effectiveness of RA Separation Using Different Biosurfactants

Within the bulk liquid phase, RA molecules are effectively entrapped by the abundant binding sites provided by the biosurfactants. Subsequently, the resulting biosurfactant and RA complexes migrate toward and spontaneously adsorb onto the ascending air and water interfaces, predominantly driven by hydrophobic interactions and synergistic interfacial thermodynamics. During the ascending foam phase, the effective separation of RA from the bulk extractant is realized through the continuous entrainment of these complexes at the bubble interfaces and within the interstitial liquid confined in the Plateau borders.

However, continuous increments in biosurfactant concentration beyond an optimal threshold did not yield proportional benefits. Instead, excessive collector molecules competitively occupied the limited interfacial area, while concurrently promoting the formation of micelles in the bulk solution. This micellar solubilization severely reduced the effective collisions and binding probability between residual trace RA and the gas interfaces, thereby exerting a regressive effect on both RA recovery and surface excess (*Γ*). This hydrodynamic trade-off is fundamentally rooted in the competitive balance between gravitational drainage and capillary suction within the foam matrix. As the biosurfactant concentration increases beyond the optimal threshold, the resulting low interfacial tension and high interfacial viscoelasticity rigidify the gas–liquid boundaries. This interfacial immobilization, coupled with the increased interstitial fluid viscosity, severely restricts gravity-driven liquid drainage. Simultaneously, the elevated surfactant concentration prevents bubble coalescence, yielding a smaller average bubble size. These smaller bubbles form narrower Plateau borders, which exponentially increase hydraulic resistance and capillary suction, effectively locking liquid within the foam nodes. Consequently, a substantial volume of unselective bulk liquid is trapped and entrained into the foamate. This increased liquid holdup dilutes the biosurfactants enriched at the gas–liquid interface, ultimately causing the observed drop in the macroscopic enrichment ratio [34].

The solution pH exerts a profound and deterministic influence on the efficacy of foam fractionation. It primarily dictates the ionization states, protonation degrees, and structural conformations of both the collector and the target molecules, thereby modulating their electrostatic interactions and interfacial adsorption capabilities [35,36]. The fundamental mechanisms driving these pH-dependent variations are deeply rooted in electrostatic interactions, as elucidated by the Zeta potential (ζ) trajectories in Figure 4k–o. Specifically, the solution pH radically alters the surface charge density of the biosurfactants. Under highly acidic conditions (lower pH ranges), intense protonation leads to the competitive occupation of binding sites by excessive hydrogen ions. This phenomenon leaves scarce vacant adsorption sites for RA and induces unfavorable electrostatic profiles, culminating in substandard surface excess and diminished RA recovery. Additionally, the severely acidic environment destabilizes the foam matrix, causing a sharp decrease in liquid holdup and an artificial spike in the enrichment ratio due to rapid film drainage. Conversely, under higher pH conditions, both the RA molecules and the biosurfactant collectors become heavily deprotonated and bear strong negative charges, which is visually corroborated by the sharp drop in zeta potential values (Figure 4k–o). The resulting fierce electrostatic repulsion between them severely hinders the formation and stability of the biosurfactant and RA complexes, triggering a drastic collapse in interfacial adsorption efficiency and an inevitable plunge in the overall recovery percentage. Thus, optimizing pH to perfectly balance these electrostatic attraction and repulsion forces remains paramount for maximizing foam fractionation efficiency.

This signal attenuation implies that higher pH environments restrict the exposure of non-polar patches on the protein structure, thereby lowering surface hydrophobicity. Such diminished hydrophobic characteristics inherently weaken the physical affiliations linking RA to WSP. Consequently, the capacity of these molecular complexes to effectively adsorb onto rising gas–liquid interfaces is severely impaired. Ultimately, these dynamic separation outcomes correspond seamlessly with earlier measurements regarding WSP hydrophobic tendencies and fundamental foaming attributes (Figure 3e).

### 4.3. Box–Behnken Design Optimization of Operational Parameters

Mechanistically, the synergistic coupling between protein concentration and environmental acidity is fundamentally logical. The initial WSP dosage dictates the absolute quantity of structural binding sites available for rosmarinic acid entrapment, while the environmental pH strictly governs the protonation state and electrostatic accessibility of these specific macromolecular pockets. Furthermore, the profound interactive effect between pH and gas flow rate is deeply rooted in interfacial hydrodynamics. The solution acidity drastically alters the viscoelasticity and surface tension of the resulting proteinaceous film, directly dictating how effectively the interstitial fluid resists the upward kinetic momentum generated by varying gas flow rate. Ultimately, this accurate predictive capability proves highly beneficial for guiding future scale-up and optimization of this specific fractionation process.

### 4.4. Mechanisms Underlying the Complexation Between RA and WSP Collector

To fully elucidate how the biosurfactant captures the target phytochemical and transports it to the foam interface, a comprehensive multi scale investigation encompassing morphological, spectroscopic, thermodynamic, and computational analyses was conducted. These morphological transformations suggest RA interaction fundamentally altered WSP’s surface properties and dispersion behavior, prompting further elemental investigation. These precise compositional variations present compelling evidence for active chemical binding. The quantifiable data point toward alterations in surface charge profiles and direct functional group coupling during the association process. This targeted shifting of the amide I signal, alongside modifications to the overall peak contour, serves as robust proof that the ligand initiated spatial rearrangements within the structural backbone of WSP [37]. Such a pronounced blue shift typically denotes an intensification of hydrogen-bonded networks or varying dipole moments. Ultimately, these spectral dynamics reflect a ligand-driven conformational stabilization, which frequently enhances surface activity in foaming systems.

Alterations within these emission spectra signify critical modifications to the tertiary configuration, which may encompass ligand attachment, domain dissociation, or partial structural relaxation [38]. This characteristic attenuation firmly verifies the successful creation of an RA-WSP aggregate. More importantly from a separation perspective, the observed bathochromic shift indicates that upon ligand binding, the macromolecular architecture relaxed and unfolded. This structural unfolding exposes previously shielded nonpolar amino acid residues to the aqueous environment, thereby dramatically increasing the surface hydrophobicity of the complex. This newly acquired hydrophobic character acts as the primary thermodynamic engine driving these aggregates toward the rising gas bubbles during the fractionation cycle.

Generally, emission attenuation originates from either kinetic collisions (dynamic pathways) or the generation of non-fluorescent ground-state assemblies (static pathways) [39,40]. Such elevated magnitudes prove definitively that the observed phenomenon is static in nature, stemming directly from stable physical binding rather than random collisions. This mathematical result implies exactly one primary attachment site exists per molecule during this specific separation process.

Assessing enthalpy (Δ*H*) alongside entropy (Δ*S*) reveals the principal molecular forces governing the system. Such a metric signifies a highly spontaneous thermodynamic tendency for the surfactant to associate with the target species [41]. Concurrently, determining both Δ*H* and Δ*S* as negative quantities highlights the specific intermolecular dynamics at play. It implies that hydrogen bond networks, operating synergistically with van der Waals interactions, act as the predominant factors facilitating the assembly and subsequent structural stabilization of the protein collector agglomerate.

These significantly negative energetic parameters corroborate preceding empirical data, confirming the generation of highly stable physical complexes. Synthesizing these predictive computational metrics with the preceding fluorometric and infrared characterizations yields a perfectly unified mechanistic model. The computational observation of extensive hydrogen bonding and hydrophobic clustering exactly mirrors the negative enthalpy and entropy values derived from our macroscopic thermodynamic calculations. Together, they confirm that the spontaneous creation of this targeted agglomerate relies upon a synergistic interplay of noncovalent drivers, which ultimately modifies the protein conformation to optimize bubble attachment efficiency.

### 4.5. RA Product Analysis

Phenolic compounds, encompassing the target molecule recovered in this study, typically exert their protective biological effects by neutralizing reactive oxidative species [42]. Consequently, recovering this specific phenolic agent via bubble-assisted separation utilizing the WSP complex significantly preserves and enhances its functional bioactivity. Such dramatic improvements in biochemical functionality are primarily attributable to the substantial elimination of interfering crude impurities during the targeted enrichment process, yielding a product of substantially higher purity.

HPLC analysis of the protein-depleted foamate (Figure S5) revealed that the peak intensity of RA increased by 2.9-fold (from approximately 38 mAU to 110 mAU) compared to the raw extract. This direct quantitative evidence confirms that the overall enhancement in the antioxidant activity of the foamate is predominantly governed by the physical concentration of RA. The additional antioxidant improvement beyond this 2.9-fold RA enrichment is likely attributed to the auxiliary radical-scavenging properties of the residual WSP matrix and potential synergistic WSP-RA interactions.

## 5. Conclusions

In this study, a highly efficient, environmentally benign foam fractionation process was successfully developed for the targeted recovery of rosmarinic acid (RA) from Perilla leaves. By systematically screening various classes of biosurfactants, whey soy protein (WSP) was identified as the most robust dual-functional frother and protein collector. Under optimized operational conditions (850 mg/L WSP, pH 2.5, and a gas flow rate of 470 mL/min), the process achieved an exceptional RA recovery rate of 93.08% and an enrichment ratio of 1.81.

The superior macroscopic separation efficiency of WSP was thoroughly validated at the molecular level. Spectroscopic and computational analyses confirmed the spontaneous, static quenching complexation between RA and WSP components (KTI and BBI). This interaction is strictly governed by a synergistic combination of non-covalent forces, primarily hydrogen bonding, van der Waals forces, π-stacking, and salt bridges. These bindings induce targeted conformational rearrangements within the protein backbone, exposing hydrophobic domains that drastically enhance thermodynamic affinity for the gas–liquid interface. Furthermore, the foam fractionation process successfully preserved and concentrated the bioactivity of the target molecule, with the recovered RA exhibiting up to a 3.6-fold enhancement in antioxidant capacity compared to the crude aqueous extract.

Ultimately, this research bridges fundamental interfacial science with applied green chemistry. It provides critical mechanistic insights into how distinct biosurfactant architectures dictate separation efficiency, offering a scalable and sustainable framework for the industrial purification of non-amphiphilic bioactive compounds. While this study successfully establishes thermodynamic and operational feasibility in a batch system, the transition to industrial application requires further engineering validation. Future studies will systematically investigate long-term continuous operations and multi-batch cyclic stability to fully assess the robust scalability of this process.

## Figures and Tables

**Table 1:** Binding parameters and thermodynamic parameters for the WSP–RA system.

*T* (K)	*K_SV_* (10^3^ M^−1^)	*K*q (10^12^ M^−1^s^−1^)	*R* ^2^	*n*	∆*G* (kJ/mol)	∆*H* (kJ/mol)	∆*S* (J/mol)
298.15	14.3	14.3	0.9946	1.226	−27.67	−104.34	−257.15
308.15	2.54	2.54	0.9962	1.088	−25.10		
318.15	1.83	1.83	0.9911	0.9295	−22.53		

**Table 2:** Type of interaction between WSP and RA.

	Binding Energy (kcal/mol)	Hydrogen Bond Residues	Hydrophobic Interaction Residues	Salt Bridge Interaction Residues	π-Cation Interactions Residues
KTI + RA	−3.52	ASP-1, TYR-62, ARG-63	PHE-2, ILE-64, ARG-65, ALA-68	ARG-65	HIS-71
BBI + RA	−4.18	SER-4, PRO-61	LYS-6, HIS-33, TYR-59, PRO-64	LYS-6	

## Data Availability

The original contributions presented in this study are included in the article/Supplementary Materials. Further inquiries can be directed to the corresponding author.

## Supplementary Materials

The following supporting information can be downloaded at https://www.mdpi.com/article/10.3390/foods15142525/s1. S1. Experimental apparatus and operation procedure of foam fractionation, Figure S1: Schematic diagram of the foam fractionation apparatus; S2. Form Property [43,44]; S3. Determination of surface excess and liquid holdup; S4. Fluorescence measurements [20]; S5. RA product analysis [45,46]; S6. Response surface methodology determine the relationship between factors for form fractionation of RA, Table S1: BBD matrix and experimental results, Table S2: ANONA results of response surface model for recovery percentage (R) of RA, Table S3: ANONA results of response surface model for enrichment ratio (E) of RA; S7. EDS and element mapping, Figure S2: EDS and element mapping for WSP(a) and WSP-RA(b), Figure S3: Size Histograms of WSP(a) and WSP-RA(b); S8. RA product analysis, Figure S4: Antioxidant activities of RA sample from the extract and after the foam fractionation, Figure S5: HPLC profile RA concentration in the three sample were 100 mg/L; S9. Estimate technical and economic outlook, Table S4: Preliminary techno-economic and operational estimations for scaled-up WSP-mediated foam fractionation.
