# Supplementary material for: Green Recovery of Rosmarinic Acid via Whey Soy Protein-Mediated Foam Fractionation: Molecular Mechanisms and Enhanced Antioxidant Activity
Source: Foods. 2026 Jul 16;15(14):2525. doi: 10.3390/foods15142525 (PMC13407702; doi:10.3390/foods15142525)
Supplement: Supplementary file 1 [file foods-15-02525-s001.zip › foods-4384891-supplementary/foods-4384891-supplementary update 13.07.pdf]

Supplementary Information

Green Recovery of Rosmarinic Acid via Whey Soy Protein-Mediated Foam Fractionation: Molecular Mechanisms and Enhanced Antioxidant Activity

Yanfei Li, Run Yang, Hongjie Xiang, Zhirong Zhang, Zhijun Zhang, Nan Hu*

School of Chemistry and Chemical Engineering, North University of China, No.3 Xueyuan Road, Jiancaoping District, Taiyuan 030051, China

Corresponding author: Nan Hu*

Tel.: +86 0351-3945368; **Fax:** +86 0351-3945368.

E-mail address: nanhu@nuc.edu.cn

Table of Contents

S1 Experimental apparatus and operation procedure of foam fractionation

S2 Foam property

S3 Determination of surface excess and liquid holdup

S4 Fluorescence measurements

S5 RA product analysis

S6 Response surface methodology determine the relationship between factors for foam fractionation of RA

S7 EDS and element mapping

S8 RA product analysis

S9 Estimated technical and economic outlook

S1 Experimental apparatus and operation procedure of foam fractionation

S1.1 Experimental apparatus

Fig. S1 depicts a schematic diagram of the experimental apparatus of foam fractionation. The foam fractionation column was a vertical column and it was constructed by the plexiglass and had 1000.0±2.0 mm in height and 40.0±1.0 mm in inner diameter.

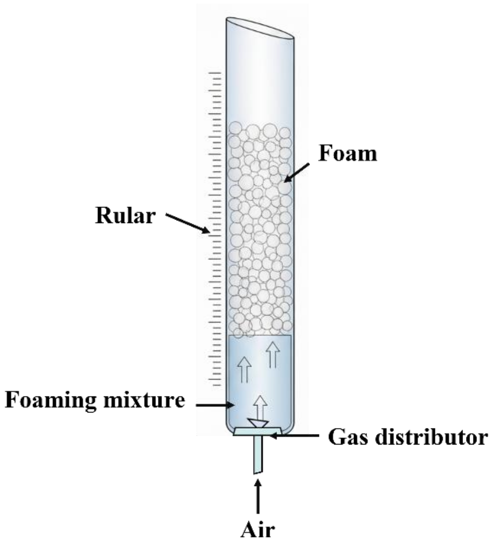

Fig. S1 Schematic diagram of the foam fractionation apparatus.

S1.2 Operational procedures

Foam fractionation was conducted in a continuous mode, and its operational procedure was shown in Figure S1 and described below. The rosmarinic acid (RA) extracts with varying biosurfactants concentrations and pH were continuously introduced into the column through the junction of the liquid and foam phases at a constant flow rate using a peristaltic pump (BT100-2J, Baoding Lange Constant Flow Pump Co., Ltd., China). The equilibrium at the foam-liquid interface was regulated by adjusting the valve at the bottom of the column to discharge the residual solution at a

constant flow rate. Compressed air was supplied to the column via an air compressor (ACO-004, Rao Ping County Xingcheng Mechanical & Electrical Aquarium Supplies Co., Ltd., China) through the gas bottle, moistening bottle, and gas distributor. The gas velocity was controlled using a rotameter (LZB-3, Tianjin Hedong Wuhuan Instrument Factory, China). As the foam layer rose, it flowed into the collector located at the top of the column for foam collection and defoaming. The experiment was conducted at room temperature. After the foam fractionation process, the foamate was freeze-dried (Scientz-10 N, Ningbo Scientz Biotechnology Co. Ltd., China), and the obtained powder was considered as the surfactant-RA complex.

S2 Foam property

The foam property tests were conducted by the modified Ross-Miles method [43,44]. Fig. S1 presents the schematic diagram drawing of the experimental setup for foamability and foam stability. 200 mL of RA extracting solution and biosurfactants was added into a glass column (diameter: 4 cm; height: 50 cm) fitted with a glass porous plate placed at the base of the column. Air was injected through the plate at the bottom of the solutions at the flow rate of 300 mL/min to generate foam for 60 s. The initial foam height (h_f) was considered as a criterion for foamability and recorded as long as the foam remained constant. The time for foam height to reduce to half of its initial height was recorded as the half life ($t_{1/2}$), which gave an indication of foam stability. The foam morphology (i.e. structure) was captured by a microscopic camera (CKX53, Olympus Corporation Co.).

S3 Determination of surface excess and liquid holdup

S3.1 Determination of surface excess of RA

When the foam-solution interface and RA concentration in the residual solution remained constant, a steady state was achieved during continuous foam fractionation the continuous foam fractionation. At this point, the surface excess of RA at the bubble surface was calculated using Eq. (S1) based on the material balance.

$$\Gamma = \frac{\Phi}{A} = \frac{C_f V_f - C_e V_f}{A} \quad \text{(S1)}$$

where Γ (mg/m^2) is the surface excess; Φ (mg/s) is the adsorbed mass flux; C_f(mg/m^3) and C_e (mg/m^3) are the RA concentrations in the foamate and the liquid phase, respectively; V_f (m^3/s) is the volumetric flux of the foamate; A (m^2/s) is the bubble surface area flux, which is determined using Eq. (S2).

$$A = \frac{6V_g}{D_{Sm}} \quad \text{(S2)}$$

where V_g (m^3/s) is the air flowrate; D_{Sm} (m) is the Sauter mean bubble diameter in the foam phase.

Sauter mean bubble diameter was determined using Eq. (S3) and analyzed with the software (Scion Image) from digital photographs taken through the column wall. The photographs were taken at the center of foam phase. In each photograph, at least 200 bubbles are measured.

$$D_{Sm} = \frac{\sum_{i=1}^{k} D_i^3}{\sum_{i=1}^{k} D_i^2} \quad \text{(S3)}$$

where D_i is the ith bubble diameter and k is the number of bubbles.

S2.2 Determination of the liquid holdup of the foam out of the column

At the steady state, the liquid holdup of the foam out of the column was calculated by Eq. (S4).

$$\varepsilon_{out} = \frac{Q_0 - Q_r}{Q_0 - Q_r + Q_g} \quad \text{(S4)}$$

where Q_0, Q_r and Q_g (mL/min) are the volumetric velocities of the feeding solution, the residual solution and the air flowrate, respectively.

S4 Fluorescence measurements

Fluorescence spectra were recorded utilizing a Gangdong F-320 fluorescent spectrophotometer (China). The intrinsic fluorescence was assessed at an excitation wavelength of 350 nm, with emission spectra captured from 380 to 450 nm using 5.0 nm slit widths for both excitation and emission. For extrinsic fluorescence, involving the ANS probe, a mixture was prepared by combining 4 mL of protein sample with 15 μL of an 8.0 mM ANS solution. The excitation wavelength was maintained at 370 nm, and emission spectra were scanned from 350 to 650 nm with a 5.0 nm slit width [20].

S5 RA product analysis

S5.1 DPPH scavenging activity

DPPH radical scavenging activity was determined according to the method reported by [45] with some modifications. In brief, 0.3 mL of sample was mixed with 2.7 mL of 60 μM DPPH radical solution in methanol. The mixture was then shaken vigorously and incubated for 30 min at room temperature in the dark. The absorbance at 518 nm was measured using a UV-visible spectrophotometer. Results were expressed as percentage inhibition of the DPPH radical. Percentage inhibition of the DPPH radical was calculated as follows:

$$\text{DPPH scavenging activity (\%)} = \frac{A_{\text{control}} - A_{\text{sample}}}{A_{\text{control}}} \times 100\% \qquad \text{(S5)}$$

where A_{sample} is the absorbance of the test sample (DPPH solution plus test sample); and A_{control} is the absorbance of the control (DPPH solution without sample).

S5.2 ABTS scavenging activity

ABTS assay was carried out using the procedure used in the previous study [46] ABTS radical cation was prepared by mixing 7 mM ABTS stock solution with 2.45 mM potassium persulfate in equal quantities, and the mixture was left in dark at room temperature for 12 h until the reaction was completed and the absorbance was stabled. The radical cation formed is further diluted in ratio (1:1) with ethanol to adjust the absorbance value to 0.700 at 734 nm using UV-Vis spectrophotometer. A 5 μL of sample was mixed with 4000 μL of ABTS• solution and allowed to stand in the dark for 2 h at room temperature. The absorbance was determined at 734 nm using a UV-Vis Spectrophotometer. All the measurements were carried out at least three times.

Percentage inhibition of $ABTS^{+}$ radical was calculated for sample using the following equation:

$$\text{ABTS scavenging activity (\%)} = \frac{A_{\text{control}} - A_{\text{sample}}}{A_{\text{control}}} \times 100\% \tag{S6}$$

where A_{sample} is the absorbance of the test sample (ABTS solution plus test sample); and A_{control} is the absorbance of the control (ABTS solution without sample).

S5.3 Protein Removal and HPLC Analysis of Rosmarinic Acid

To quantify the exact enrichment of rosmarinic acid (RA) without interference from the protein carrier, the collected foamate was adjusted to pH 4.4 using 0.1 M HCl to induce the isoelectric precipitation of WSP. The mixture was centrifuged at 8,000 rpm for 15 min at 4 °C to completely remove the protein pellet. HPLC analysis of the obtained supernatant and the initial crude extract.

Chromatographic separation utilized a Zorbax Eclipse XDB-C18 reversed-phase column (150 mm × 4.6 mm, 5 μm particle size, 35 °C). The eluent, a combination of 0.1% acetic acid aqueous solution (mobile phase A) and methanol (mobile phase B), was filtered through a 0.22 μm porous nylon membrane and degassed under vacuum conditions. A 10 μL injection volume was eluted at a flow rate of 1 mL/min, with detection conducted at 280 nm.

S6. Response surface methodology determine the relationship between factors for foam fractionation of RA.

Table S1 BBD matrix and experimental results

Run	Independent variables			Response variable	
	Concentration of the collector, *A* (mg/L)	pH, *B*	Rate of gas velocity, *C* (mL/min)	Recovery percentage (R_{RA})	Enrichment ratio (E_{RA})
1	600	4	400	83.755	2.62604
2	1000	4	400	76.712	2.4022
3	600	3	500	87.8462	1.9221
4	800	3	400	90.2249	1.89022
5	800	2	500	92.9882	1.86022
6	1000	2	400	87.2308	2.08894
7	800	3	400	92.6006	1.84112
8	600	2	400	81.5716	2.29022
9	1000	3	500	89.1746	1.89022
10	800	4	500	79.2923	2.34022
11	800	3	400	90.2145	1.88022
12	800	2	300	73.7592	2.55884
13	600	3	300	80.6538	2.14022
14	1000	3	300	86.3935	1.98221
15	800	3	400	91.7751	1.8734
16	800	3	400	89.7071	1.97332
17	800	4	300	80.1231	2.34022

Table S2 ANONA results of response surface model for recovery percentage (*R*) of RA

Source	Sum of squares	Degree of freedom	Mean Square	*F*-value	*P*-value
**Model**	531.46	9	59.05	17.27	0.0005
A	4.04	1	4.04	1.18	0.3131
B	30.68	1	30.68	8.97	0.0201
C	100.62	1	100.62	29.43	0.0010
AB	40.34	1	40.34	11.80	0.0109
AC	4.86	1	4.86	1.42	0.2718
BC	100.60	1	100.60	29.42	0.0010
A^2	17.79	1	17.79	5.20	0.0566
B^2	179.63	1	179.63	52.54	0.0002
C^2	33.77	1	33.77	9.88	0.0163
**Residual**	23.93	7	3.42		
Lack of Fit	17.93	3	5.98	3.98	0.1077
Pure Error	6.01	4	1.50		
**Cor Total**	555.39	16			

$P < 0.01$ indicates highly significant, $0.01 < P < 0.05$ indicates significant, and $P > 0.05$ indicates not significant.

Table S3 ANONA results of response surface model for enrichment ratio (*E*) of RA

Source	Sum of squares	Degree of freedom	Mean Square	*F*-value	*P*-value
**Model**	1.05	9	0.1166	15.04	0.0009
A	0.0473	1	0.0473	6.10	0.0429
B	0.1036	1	0.1036	13.37	0.0081
C	0.1272	1	0.1272	16.41	0.0049
AB	0.0001	1	0.0001	0.0164	0.9017
AC	0.0040	1	0.0040	0.5131	0.4970
BC	0.1220	1	0.1220	15.74	0.0054
A^2	0.0301	1	0.0301	3.88	0.0896
B^2	0.5943	1	0.5943	76.67	< 0.0001
C^2	0.0002	1	0.0002	0.0308	0.8657
**Residual**	0.0543	7	0.0078		
Lack of Fit	0.0446	3	0.0149	6.13	0.0561
Pure Error	0.0097	4	0.0024		
**Cor Total**	1.10	16			

S7. EDS and element mapping

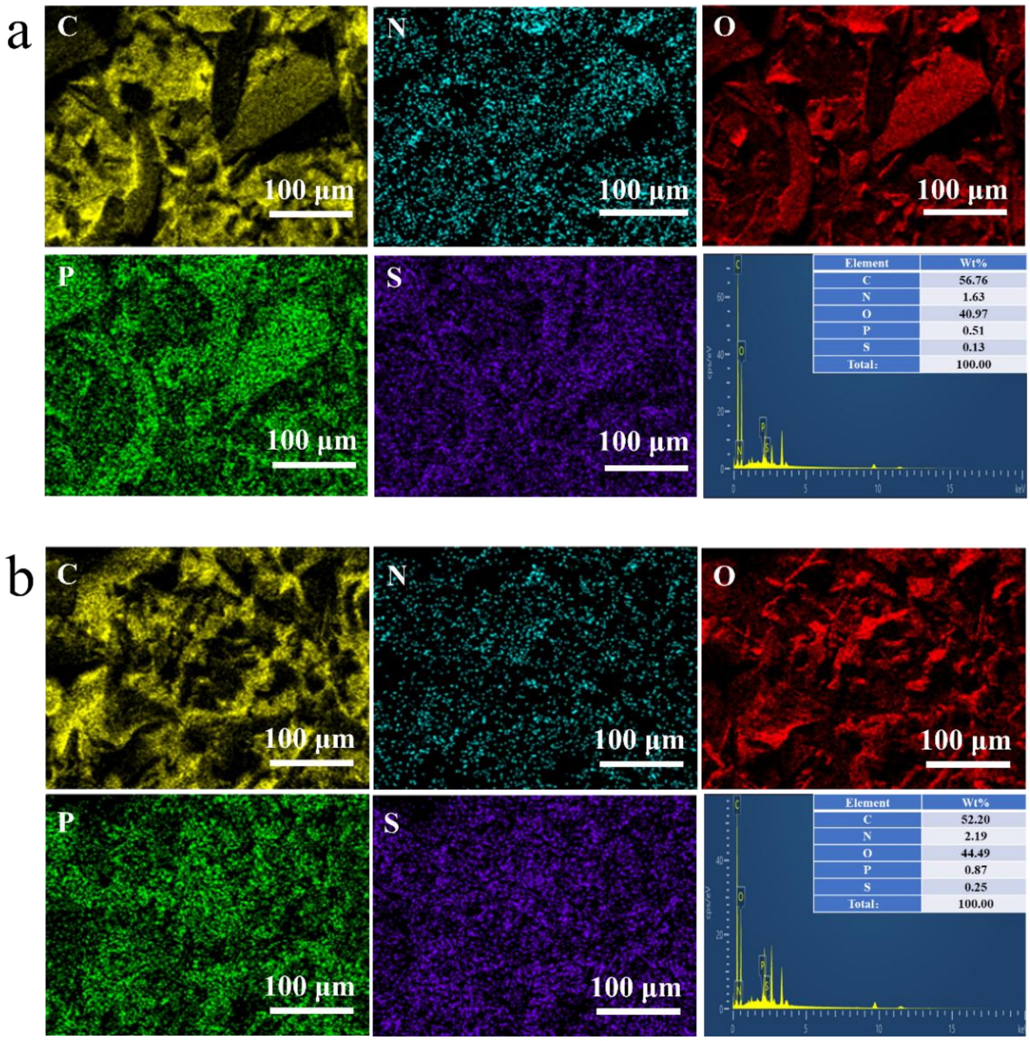

Fig. S2 EDS and element mapping for WSP (a) and WSP-RA (b)

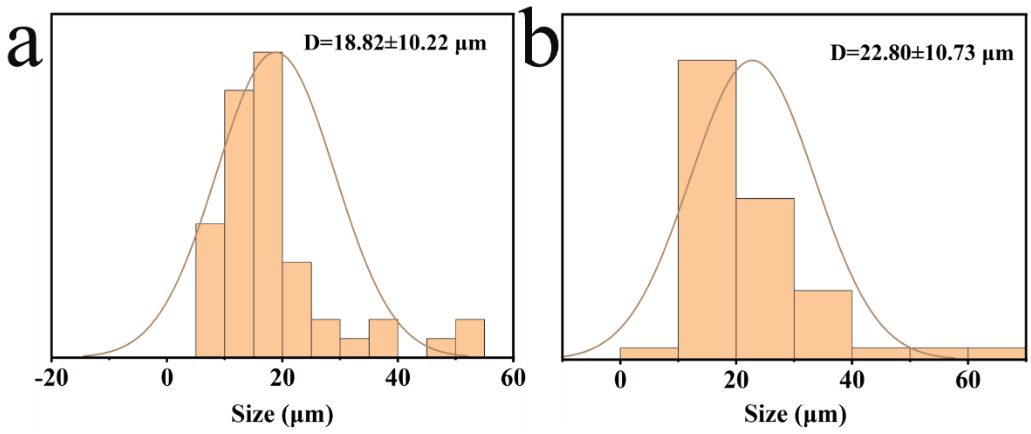

Fig. S3 Size histograms of WSP (a) and WSP-RA (b)

S8. RA product analysis

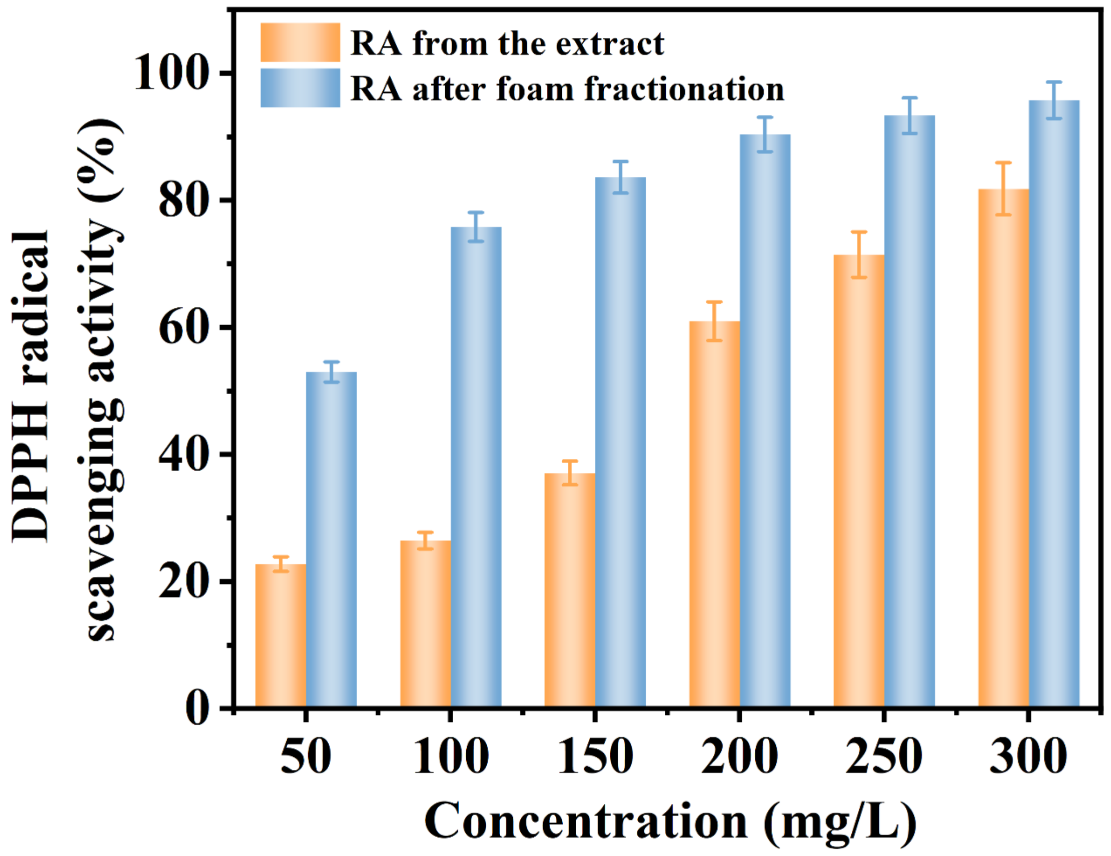

Fig. S4. Antioxidant activities of RA samples from the extract and after the foam fractionation.

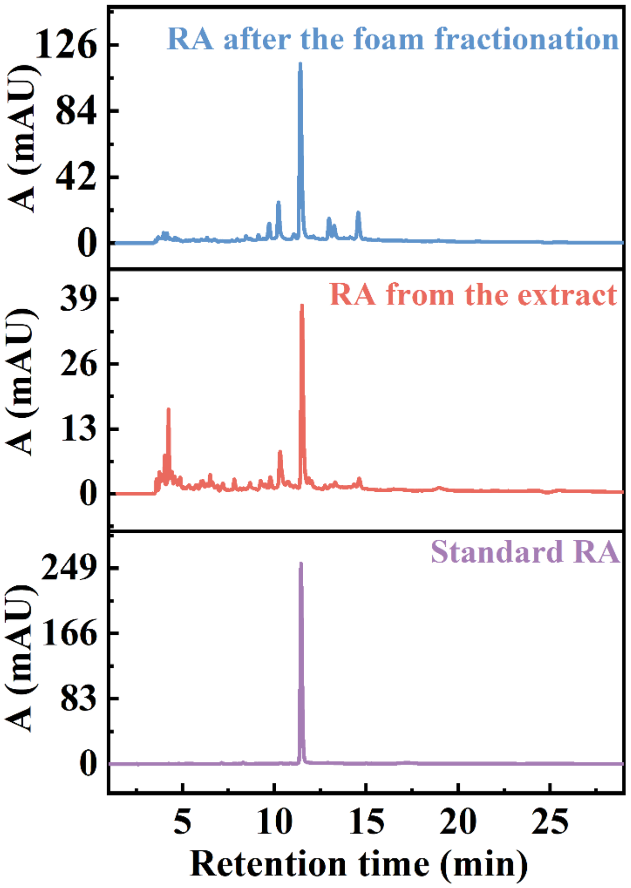

Fig. S5 HPLC profiles RA concentrations in the three samples were 100 mg/L.

S9 Estimated technical and economic outlook

The intrinsic operational characteristics of the WSP-mediated foam fractionation system present significant techno-economic advantages for industrial scale-up. By replacing highly purified commercial proteins and petroleum-derived synthetic surfactants with WSP—an abundantly available, low-value agricultural byproduct—material costs are drastically minimized. Furthermore, the process operates entirely at ambient temperature and pressure. By relying solely on low-pressure air compression for bubbling, it completely eliminates the massive thermal energy requirements of distillation and the consumption of expensive, hazardous volatile organic solvents. A preliminary estimation of processing costs, energy consumption, and expected industrial throughput is summarized in Table S4.

Table S4. Preliminary techno-economic and operational estimations for scaled-up WSP-mediated foam fractionation

**Parameter**	**Estimated Value / Characteristic**	**Advantage / Justification**
**Material Cost (Collector)**	Very Low (< \$1–2/kg for bulk WSP)	Direct upcycling of agricultural soybean byproduct, significantly cheaper than purified proteins or commercial surfactants.
**Solvent Consumption**	Zero organic solvents	Uses exclusively aqueous solutions; eliminates costs for solvent purchase, recovery, and hazardous waste disposal.
**Energy Consumption**	~0.1–0.5 kWh/m^3 (ambient aeration)	Avoids thermal energy (heating/cooling) and high-pressure pumping, relying only on basic air compressors and feed pumps.
**Expected Throughput**	50–100 L/h (for a standard continuous column, Ø 10–20 cm)	Highly efficient as a rapid, high-throughput primary volume-reduction and pre-concentration step.

Supplementary References

20. Wang, Y.; Wang, S.; Li, R.; Wang, Y.; Xiang, Q.; Li, K.; Bai, Y. Effects of combined treatment with ultrasound and pH shifting on foaming properties of chickpea protein isolate. Food Hydrocolloid. 2022, 124, 107351.

43. Zhao, J.; Torabi, F.; Yang, J. The synergistic role of silica nanoparticle and anionic surfactant on the static and dynamic CO_2 foam stability for enhanced heavy oil recovery: An experimental study, Fuel. 2021, 287, 119443.

44. Farhadi, H.; Riahi, S.; Ayatollahi, S.; Ahmadi, H. Experimental study of nanoparticle-surfactant-stabilized CO_2 foam: Stability and mobility control, Chem. Eng. Res. Des. 2016, 111 449-460.

46. Liu, J.; Wu, G.; Yang, J.; He, C.; Xiong, H.; Ma, Y. Abalone visceral peptides containing cys and tyr exhibit strong in vitro anti-oxidant activity and cytoprotective effects against oxidative damage. Food Chem. X. 2023. 17, 100582.

47. Hussen, E.M.; Endalew, S.A. In vitro antioxidant and free-radical scavenging activities of polar leaf extracts of vernonia amygdalina. BMC Complement. Med. 2023. 23, 146.
